# Machine Learning Approach to Model Physical Fatigue during Incremental Exercise among Firefighters

**DOI:** 10.3390/s23010194

**Published:** 2022-12-24

**Authors:** Denisse Bustos, Filipa Cardoso, Manoel Rios, Mário Vaz, Joana Guedes, José Torres Costa, João Santos Baptista, Ricardo J. Fernandes

**Affiliations:** 1 Associated Laboratory for Energy, Transports and Aeronautics, Faculty of Engineering, University of Porto, 4200-465 Porto, Portugal; 2 Centre of Research, Education, Innovation and Intervention in Sport, CIFI2D, Faculty of Sport, University of Porto, 4200-450 Porto, Portugal; 3 Porto Biomechanics Laboratory, Faculty of Sport, University of Porto, 4200-450 Porto, Portugal; 4 Associated Laboratory for Energy, Transports and Aeronautics, Faculty of Medicine, University of Porto, 4200-319 Porto, Portugal

**Keywords:** fatigue estimation, physiological signals, classification algorithms, health and safety

## Abstract

Physical fatigue is a serious threat to the health and safety of firefighters. Its effects include decreased cognitive abilities and a heightened risk of accidents. Subjective scales and, recently, on-body sensors have been used to monitor physical fatigue among firefighters and safety-sensitive professionals. Considering the capabilities (e.g., noninvasiveness and continuous monitoring) and limitations (e.g., assessed fatiguing tasks and models validation procedures) of current approaches, this study aimed to develop a physical fatigue prediction model combining cardiorespiratory and thermoregulatory measures and machine learning algorithms within a firefighters’ sample. Sensory data from heart rate, breathing rate and core temperature were recorded from 24 participants during an incremental running protocol. Various supervised machine learning algorithms were examined using 21 features extracted from the physiological variables and participants’ characteristics to estimate four physical fatigue conditions: low, moderate, heavy and severe. Results showed that the XGBoosted Trees algorithm achieved the best outcomes with an average accuracy of 82% and accuracies of 93% and 86% for recognising the low and severe levels. Furthermore, this study evaluated different methods to assess the models’ performance, concluding that the group cross-validation method presents the most practical results. Overall, this study highlights the advantages of using multiple physiological measures for enhancing physical fatigue modelling. It proposes a promising health and safety management tool and lays the foundation for future studies in field conditions.

## 1. Introduction

Fatigue is a multidimensional phenomenon resulting from the combination of various factors [1,2] (e.g., time-of-day, extreme workloads, health, on-the-job and off-duty responsibilities and lifestyle [3]). In the workplace, it is associated with the inability to continue with an activity at the desired level because of mental and physical exhaustion [4]. Mental fatigue is related to decreased motivation and ability to respond to information resulting in diminished alertness and productivity [5]. On the other hand, physical fatigue can be described as the inability to maintain the physical capability to perform a task optimally and is generally the result of prolonged work tasks, adverse environmental conditions and inadequate rest breaks [6].

From an occupational and safety view, fatigue management is of utmost importance since it has major immediate and long-term implications [7], including decreased cognitive and motor abilities, reduced work efficiency and productivity and subsequent heightened risk of accidents [8]. The consequences can aggravate even further within safety-sensitive professions, such as the military, police officers and firefighters [9]. Indeed, this last group is one of the most physically demanding occupations, with a considerably high rate of on-duty fatalities worldwide [10]. Extremely hot environments, high work intensity and heavy, impermeable protective clothing and equipment all together expose firefighters to substantial cardiorespiratory and thermoregulatory stress levels. Consequently, and before unacceptable risk levels are reached, preventive and interventional measures should be taken [11].

Since physical exertion is considered the primary source of fatigue, different methods have been proposed for its estimation, such as monitoring physiological responses and the use of subjective scales [12]. To avoid subjectivity and allow continuous monitoring, wearable technology has also made major advances, facilitating the noninvasive collection of multiple physiological variables in real-time [13,14]. Accordingly, literature has evidenced that combining different physiological variables can help in more accurate physical fatigue assessments, and recent studies have addressed this multivariable approach among occupational groups [15,16,17]. Different supervised machine learning algorithms have been proposed to estimate physical fatigue within construction workers while performing simulated manual handling tasks [8,18] and regular duties in the field [19]. Equivalent approaches have been used to determine stress levels in train drivers in a high-speed rail simulator [20] and to distinguish fatigued and non-fatigued states after specific occupational activities [12,21,22]. In addition, neural networks have been applied to develop binary fatigue classifiers during manufacturing tasks [23]. 

While these studies have opened the path for automated and individual real-time physical fatigue monitoring, more work is still needed to fully explore the potential of wearables and develop generalisable methods for assessing physical fatigue [24]. Most investigations assessed fatigue-inducing tasks with workloads that were not representative of all occupational groups, especially those that are subjected to extreme physically demanding conditions, as in the case of firefighters. Furthermore, studies have predominantly focused on detecting fatigue through binary models and have not delimited various levels to understand the transition leading to a maximal exhaustion status and individuals’ physiological limits [25]. In addition, there is scarce evidence of validation procedures of these models considering the inter-subject variability and potential data imbalances [14]. As a result, the current study aims to contribute to physical fatigue assessment, evaluating a high range of exercise intensities within this occupational group using multivariable physiological monitoring and machine learning algorithms and testing different validation methods.

## 2. Materials and Methods

### 2.1. Participants

A convenience sample of twenty-four healthy individuals (18 men) from a local fire brigade participated in the current study. Their main anthropometric characteristics were: age 33.1 ± 9.5 years, body mass 76.0 ± 10.6 kg, height 173.1 ± 7.9 cm and fat mass 22.7% ± 10.6% (InBody270; InBody Co. Ltd., Cerritos, CA, USA). They were active volunteer firefighters with no history of cardiopulmonary or intestinal diseases and an absence of musculoskeletal disorders. All experiments were conducted in accordance with the Declaration of Helsinki and approved by the Ethics Committee of the University of Porto (Report 106/CEUP/2021). An informed written consent form was read and signed by all subjects involved in the trials.

### 2.2. Data Collection and Labelling

**Figure 1:**
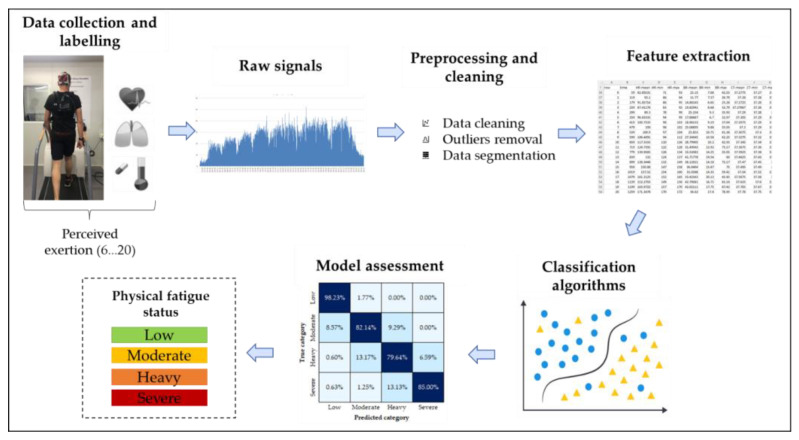
Methodology employed for implementing the physical exertion classification model.

**Figure 2:**
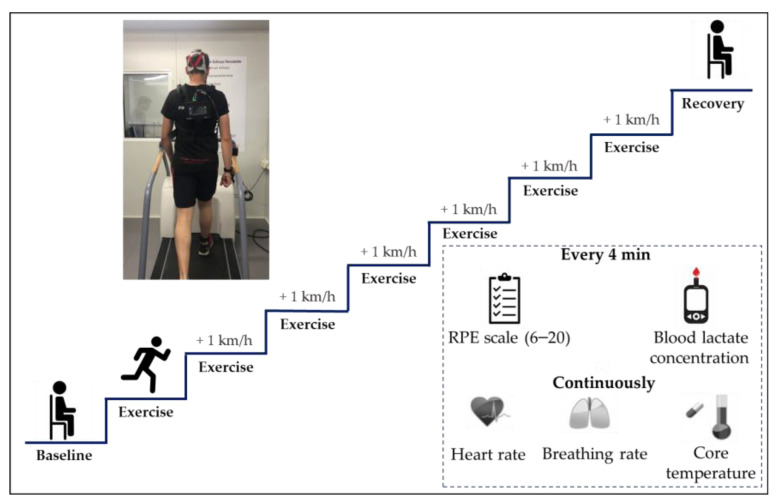
Scheme describing the implemented intermittent incremental running protocol.

The methodology employed for physical exertion monitoring and modelling is summarised in Figure 1, illustrating the steps followed from the physiological data collection, up to assessing the model’s performance. As described in Figure 2, volunteers (in light clothing, approximately 0.3 clo [26]), performed an incremental intermittent running protocol of seven stages of 4 min (with 1 km/h increments and 30 s rest periods in between) on a treadmill (T2100 treadmill; GE, Boston, MA, USA) [27,28,29] inside a climatic chamber (FITOCLIMA 25000 EC20; Aralab, Rio de Mouro, Portugal) [30]. The chamber (3.20 m × 3.20 m), which controls temperature to an accuracy of ± 0.2 °C and relative humidity of ± 5%, was set at thermoneutral conditions (24 °C and 50% of relative humidity). The participants’ initial velocity was determined according to their experience and capacity, and validated in a previous bout in which they ran at low intensity at that pre-defined pace [28,29].

Volunteers were familiarised with all testing procedures and equipment prior to the experimental sessions. The day before each trial, an explanation of the risks and benefits of participating was provided, and the informed consent form was signed by the volunteers. Next, a medical examination took place to ensure their aptness to participate and complete all parts of the experiment. With the volunteers’ and the physician’s consent, a telemetric ingestible thermometer pill (e-Celsius Performance capsule; BodyCAP, Hérouville-Saint-Clair, France) was then given with the respective indication on how and when to ingest it (6 h prior to the test) [31]. During the experimental protocol, breath-by-breath respiratory gas exchange variables were measured continuously by a telemetric portable gas analyser (COSMED K5; Cosmed, Rome, Italy). It was attached to the participant’s back and placed near their body’s centre of mass to avoid relevant interferences during running [28,29]. 

Heart rate was measured at baseline and every 5 s using a heart rate monitoring belt (Garmin Edge 830; Garmin, Olathe, KS, USA) that telemetrically emitted the data to the K5 portable unit. Intraabdominal core temperature was continuously retrieved from low-frequency radio waves transmitted from the gastrointestinal capsules to an external logger (e-Viewer Performance monitor, BodyCAP, France) at 15 s intervals [31]. The rates of perceived exertion were collected at the end of each 4 min stage through direct feedback from the participants and using the 6–20 Borg scale [28]. Finally, for complementary information, capillary blood samples for blood lactate analysis were collected (Lactate Pro2; Arkay, Inc., Kyoto, Japan) from the fingertip at baseline, during the 30 s rest stages and at the 1st, 3rd, 5th and 7th min of the recovery stage [28,29,32]. They were included as indicators of the anaerobic system contribution and to validate the physical fatigue levels determined through the Borg scale.

### 2.3. Preprocessing and Cleaning

The collected data were revised to remove errant measurements from talking, coughing or signal interruptions [28,29,32]. Signals from heart rate, core temperature, breathing rate and gas exchange variables were preprocessed to consider only the data between the mean ± 3 standard deviations and posteriorly smoothed using a moving average filter [27,28,29,32]. The first three variables were selected as the main variables and therefore included in the model, since they have been previously proven as valid and reliable indicators of physical exertion and fatigue within various occupational settings [13,16]. Furthermore, they can be obtained using sensors that allow mobility, continuous monitoring and ease of wearing [13,14,17,33]. For the main and complementary measured variables, the normality of distribution was checked using the Shapiro–Wilk’s test and mean values ± SD were calculated for every stage. Pairwise multiple post hoc comparisons were conducted with Bonferroni’s correction, with the significance level set at *p* < 0.05.

### 2.4. Feature Extraction

Preprocessed data were synchronised, testing different time intervals, and various features were extracted from heart rate, breathing rate and core temperature within each considered time interval. These features, including mean, minimum, maximum, standard deviation, variance and kurtosis, calculated from 4, 2 and 1 min intervals, and baseline values (3 min average of pre-exercise values while sitting) from the variables, were evaluated to be integrated or not into the model based on their capacity to increase the model’s prediction accuracy. After testing all the alternatives (using different feature combinations to train the model), baseline, mean, minimum and maximum values per minute were included from heart rate, breathing rate and core temperature signals. In addition, the age-predicted maximum heart rate (220-age) [29], the percentage of the age-predicted maximum heart rate (calculated from the mean heart rate per minute) and personal characteristics (i.e., gender, age, weight, height, fat mass, fat-free mass and body mass index) were combined as inputs for modelling. As described in Table 1, the level of physical fatigue reported with the 6–20 Borg Scale was simplified to a 4-level scale [12,15,18], with the resulting categories (i.e., low, moderate, heavy and severe) used as ground truth for modelling. 

### 2.5. Classification Algorithms

The resulting dataset, comprising a total of 750 sets of 21 features (six from heart rate, four from breathing rate, four from core temperature and seven from personal characteristics) together with the corresponding fatigue levels, was normalised and fed to machine learning algorithms. Various supervised classification algorithms, previously used for modelling physiological variables responses [12,15,18,34,35] and many health-related purposes [36,37], were evaluated since no previous study assessed this combination of variables for firefighting applications. The tested algorithms included K-nearest neighbours, Boosted Trees (Gradient-boosted Trees, XGBoosted Trees and RUSBoosted Trees), Bagged Trees, Random Forests, Support Vector Machines with different kernel functions (linear, quadratic, cubic and Gaussian) and Artificial Neural Networks.

K-nearest neighbours is one of the simplest yet accurate classifiers that assumes that similar results are near each other and, therefore, depends mainly on measuring the distance or similarity between the unlabelled data and the training examples [38]. Boosted Trees algorithms utilise numerous weak classification trees and turn them into strong classifiers. The weight of each classification tree is in proportion to their ability to classify given labelled examples correctly. While numerous algorithms can be used for Boosted Trees implementation, the current study used Gradient-Boosted Trees, an ensemble technique able to operate with small amounts of data [39], and XGBoosted Trees, an improved extendible application of gradient-boosted machines [40,41]. RUSBoosted Trees are a hybrid approach (sampling and boosting) especially suited for cases where the classification model is built using imbalanced data. While it takes all class samples with the least labelled examples, it undersamples other classes by taking samples equal to the class with the least examples. Then, the classifier is improved iteratively based on loss function minimisation [42].

Random Forest is an ensemble of many individual tree predictors in which each tree depends on the values of a random vector sampled independently and with the same distribution of all trees in the forest [43]. Support Vector Machines are algorithms that use a probabilistic binary linear classifier to learn the structure of the data. The kernel functions transform the features into high-dimensional spaces to improve the accuracy [19,37]. With the linear kernel, the original features of the data are used. The quadratic and cubic take each feature dimension into their squared and cubic values, respectively. The Gaussian kernel uses a radial basis function to transform the features. Finally, the Artificial Neural Network algorithm is a set of connected input-output networks (one input, one or more intermediate and one output layer) in which weight is associated with each connection, and the classification is made as belonging to some discrete class based on inputs [12]. All tested algorithms were trained with their default hyperparameters for feature selection, and then they were iteratively adjusted and retrained, modifying their training hyperparameters. The final version of each algorithm was defined with the hyperparameters combination leading to the best performance. The details of these classifiers and the process of determining their hyperparameters are not reported here, as they can be consulted in various machine learning resources [37,44]. 

### 2.6. Model Assessment

To validate the capability of the trained models to accurately predict the four physical fatigue levels, 10-fold cross-validation was initially performed. This is the most commonly used method to validate supervised machine learning algorithms and has been widely employed to measure the performance of classification models using physiological signals to predict human psychophysiological states, such as stress, emotions and physical exertion [15,19,45]. In the 10-fold cross-validation, the dataset is randomly divided into ten equal-sized subsets, with nine of the ten subsets being used to train the model and the remaining subset applied to validate the performance of the trained model. The training and validation are repeated ten times so that all subsets are used for validation and the reported accuracy is the average of the ten iterations.

Furthermore, given the characteristics of the dataset, other validation methods were also examined to determine the model’s good performance. Stratified 10-fold cross-validation was applied to solve any under or overfitting of the model resulting from the imbalanced classes (e.g., participants reported low and heavy categories more than moderate and severe). This method ensures that each fold of the dataset has the same proportion of samples with each category. Finally, group cross-validation was used with 24 splits to divide the dataset by participants and explore the capability of the model to predict the physical fatigue of every volunteer separately. In the three cases, the number of correctly predicted samples or true positives from the total amount of data, the false negatives (resenting the number of predictions wrongly classified as other fatigue groups) and the false positives (referring to the number of predictions that belong to other groups and were wrongly estimated) were calculated to obtain the four performance metrics of accuracy, precision, recall and F1 score (Equations (1)–(4), respectively). The results of these metrics were compared to determine the model with the best performance.

(1) $$\mathrm{Accuracy}=\frac{\mathrm{True}\;\mathrm{positives}}{\mathrm{Total}\;\mathrm{records}}$$

(2) $$\mathrm{Precision}=\frac{\mathrm{True}\;\mathrm{positives}}{\mathrm{True}\;\mathrm{positives}+\;\mathrm{False}\;\mathrm{positives}}$$

(3) $$\mathrm{Recall}=\frac{\mathrm{True}\;\mathrm{positives}}{\mathrm{True}\;\mathrm{positives}+\;\mathrm{False}\;\mathrm{negatives}}$$

(4) $$F1\;\mathrm{score}=\frac{\mathrm{Precision}*\mathrm{Recall}}{\mathrm{Precision}+\;\mathrm{Recall}}*2$$

## 3. Results

Data collected from the 24 participants were initially preprocessed, removing approximately 13% of the records (minimum 9%, maximum 21%, median 13%). Table 2 shows the results for each measured variable during every stage of the incremental running protocol, noting the significant differences among stages (*p* < 0.05). Although not all of them were used for developing the model (heart rate, breathing rate and core temperature were used for feature extraction and RPE for labelling the physical fatigue stages), together they provided a complete view of the participants’ performance and physiological limits while validating the physical fatigue levels resulting from the Borg scale. Preprocessed records were synchronised per minute, resulting in a dataset of 750 sets of 21 features, from which 283 belonged to the low, 140 to moderate, 167 to heavy and 160 to severe levels. Various supervised machine learning algorithms were tested on this dataset, and Table 3 displays the performance metrics of these classification algorithms using the three cross-validation techniques.

Observing the accuracy, the three methods identified XGBoosted Trees (using 500 estimators, a maximum depth of individual regression estimators of five and a learning rate of 0.1) as the best classifier. Indeed, this algorithm consistently showed the best results in accuracy, recall and F1-score. Regarding precision, Gradient-boosted Trees (with 500 estimators, maximum depth of five and learning rate of 0.1) and Random Forest (with 500 estimators, maximum depth of five, and the quality of splits measured with the Gini impurity criterion) also showed good results in stratified and group cross-validation (respectively). Overall, the XGBoosted Trees algorithm outperformed all the tested algorithms and was selected for further analysis.

**Figure 3:**
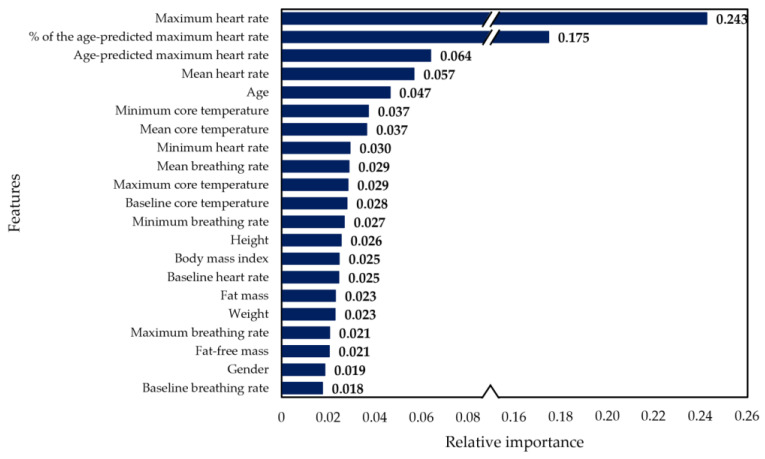
Relative importance of features for the XGBoosted Tree model.

Figure 3 presents the relative importance values obtained for each feature, with the maximum heart rate and the age-predicted maximum heart rate percentage evidencing the highest contributions to the developed model. Further analysis of the impact of these features on the model’s performance showed that excluding the maximum heart rate from the model reduced the overall accuracy to 77%, and the lowest reported individual accuracy (considering group cross-validation results) dropped to 54% (compared to 69% including the feature). On the other hand, without the percentage of the age-predicted maximum heart rate, the overall accuracy decreased to 76%, and the variability among individual accuracies increased. By excluding this feature, the lowest individual accuracy was 62% and the number of participants with accuracies under 70% went from three (including the feature) to nine.

**Figure 4:**
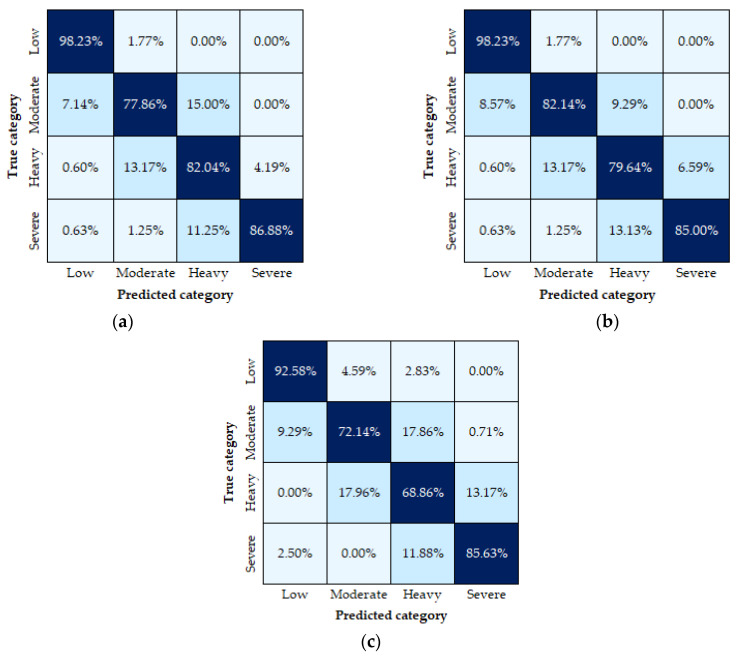
Confusion matrices: (**a**) using 10-fold cross-validation, (**b**) using stratified 10-fold cross-validation and (**c**) using group cross-validation with 24 splits.

Confusion matrices for the best accuracy algorithm are shown in Figure 4, describing the outcomes among the three cross-validation procedures. While the 10-fold and stratified cross-validation methods present less than 5% differences within the same categories, the group cross-validation displays differences of up to 10%. Nevertheless, a close examination of the three methods reveals that most misclassified cases belonged to the adjacent categories, with very few cases observed for non-adjacent categories. Interestingly, the results also report varying accuracy for the four physical fatigue categories (from 69 to 93% in the group cross-validation), with the best predictions registered in the low and severe intensities (93 and 86%, respectively). This outcome evidences the model’s ability to identify extreme physical exertion cases, which is particularly useful for field scenarios.

## 4. Discussion

Recent wearable sensors have the potential to retrieve different physiological signals in extreme conditions. However, the resulting data needs to be processed and interpreted to provide meaningful outcomes and lead to timely interventions in occupational environments. To address this gap, this study applied signal processing and machine learning techniques using physiological signals to train and validate a classification model to detect four physical fatigue levels among firefighters. Various supervised machine learning algorithms and cross-validation alternatives were explored and helped develop and validate a model with an overall performance of 88%, using 10-fold and stratified cross-validation methods, and 82%, by evaluating the predictions for each participant separately (through the group cross-validation procedure).

The current study used machine learning classifiers instead of traditional statistical methods because they do not require the manual discovery of the variables’ patterns for the different fatigue levels and are able to determine the best category by reviewing complex data without a previous view of underlying structures [46]. Machine learning focuses on prediction to then explain the causal relationships, feeding the algorithms with labelled examples such that the algorithms themselves identify the patterns within each level and adjust their parameters based on these examples [47]. In addition, deep learning approaches were not appropriate due to their lack of transparency and interpretability, their need for large datasets and their computation costs, all of which would hinder their applicability in occupational settings [48]. Therefore, different supervised classification algorithms were evaluated. The XGBoost classifier was determined to have the best performance. Consistently, supervised machine learning for binary and multiclass classification models is currently predominant among the proposed fatigue quantification approaches for occupational applications [14].

Although similar approaches exist in the literature, they mostly assessed workers during manual handling tasks [7,8,15,18], which do not reflect the firefighters’ physical demands. In their regular duties, they may be sedentary for extended periods of time, take part in training and simulated fires or be called with little to no notice into situations of danger and extreme physiological stress. To allow a complete view of their physiological responses from an unfatigued state until maximal exertion, our study applied an incremental running protocol until exhaustion in controlled conditions [27,28,29]. Training the model with the data collected using this protocol helped the algorithms to have examples of every state the subject went through until reaching a maximal exertion and being unable to continue. For field applicability, it allowed learning on the subjects’ physiological limits to intervene before they reach them. Regarding the use of the Borg scale as a prediction label, several studies have applied it for similar goals, evidencing an agreement on its usefulness and validity for physical fatigue estimation [8,12,15,18]. While it is a subjective scale, grouping it into four levels reduced the potential bias caused by reporting slightly higher or lower fatigue levels due to individual differences in understanding of the scale [15]. 

Another crucial aspect considered when developing the model was feature selection. An excessive number of features required for high-accuracy classification and monitoring could increase space and computational requirements, while irrelevant features could decrease performance [18]. Before achieving the final version of the model, alternatives on time intervals and features extracted from them were used to train different versions of the model. As a result, the features obtained per minute led to the best accuracy and were therefore included in the final model. However, as Figure 3 evidenced, the second and third most important features were the age-predicted maximum heart rate and the percentage derived from it. When eliminating only the age-predicted maximum heart rate, the variability among accuracies obtained in each fold increased and the overall accuracy decreased to 76%. While there are discrepancies in the accurateness of these two variables [49], some studies have successfully included them in their machine learning models [7] and, in the current study, they improved the consistency of results among participants and the averaged performance.

Overall, the results showed the model’s good performance (compared to similar fatigue estimation approaches [15,19]) with the 10-fold and stratified cross-validation describing equivalent scores. However, the 6% difference between them and the group cross-validation indicates the potential overfitting of the model when randomising the data. Since the model’s goal is to predict physical fatigue based on the biological individualised data, it should be able to perform well when dealing with new individuals. Therefore, dividing the data by participants helped to have a detailed view of the results obtained in each case. The interindividual accuracy variability found among subjects revealed the importance of personal data and individualised monitoring for better predictions. Concerning the categories’ accuracies, the highest was registered at the low level (93%), which might be attributed to the larger dataset for this category. For machine learning models, it is well known that a larger dataset could lead to greater performance accuracy and vice versa [44]. Of the 750 sets of features, 38% belonged to the low level, and 19%, 22% and 21% to moderate, heavy and severe levels, respectively.

Although a direct comparison with other studies is not possible because of different physiological collected data, datasets sizes, experimentation and participants, some remarks can be made of the performance of the current study against other approaches. Similar to this study, Aryal et al. [15] used a four-level exertion scale derived from the Borg scale as labels and developed a fatigue classification model based on a decision tree algorithm. However, unlike the current study, they used signals from electroencephalography, multiple infrared temperature sensors on the face and heart rate collected from 12 construction workers during simulated construction tasks and grouped in 2 min buffers. By performing 10-fold cross-validation, they obtained an 82.6% accuracy, compared to the 88% on the current study, with the most notorious differences observed in the accuracies for the low and severe intensities (87% and 82% reported by them and 98% and 87% obtained in this study), which evidence our model’s potential to predict extreme fatigue scenarios and its good performance in comparison to current literature.

Alternatively, Pluntke et al. [50] and Kupschick et al. [51] used machine learning algorithms applied to firefighters’ data. The first study used 1 min window features from heart rate variability as inputs for a decision tree algorithm to distinguish between stressed and non-stressed states [50] and, in contrast, the latter used the Borg scale but simplified it to a two-point scale to classify low strain (6–10) and high strain (15–20) [51]. They used similar features from personal characteristics, core temperature and heart rate but applied a Support Vector Machine algorithm as the classification method. In both cases, sample sizes were comparable to the current study’s (27, 22 and 24, respectively) and accuracies using the same cross-validation method were also equivalent (88%, 85.8% and 88%, respectively). Although there were differences in the considered fatigue levels and reported metrics, consistent results were observed in the highest physical fatigue level, with Pluntke et al. [50] reporting a precision of 92% and recall of 82% (compared to 87% and 95% from this study) and Kupschick et al. [51] describing a 90% accuracy in their high strain category (compared to 87% achieved in this study). This comparability of results confirms the contribution and prediction capability of the model of our study, which uses equivalent features but classifying four physical fatigue levels.

Despite the successful model implementation, the current work had some limitations. First, although the trials satisfactorily captured the gradual increment in physiological responses through each delimited physical fatigue level, they were conducted in controlled conditions with a low mental workload and thermoneutral environment. Additional physical and thermal burdens were not considered since they would have accelerated the transition to maximal exhaustion. However, during their regular duties, firefighters can be subjected to prolonged physically and mentally demanding activities and adverse climatic conditions. Hence, future studies will aim to repeat the trials under different controlled environmental conditions and in real settings, during simulated or real fires, to assess the model’s performance under those situations. Furthermore, the current study measured breathing rate and core temperature from a portable gas unit and a thermometer capsule (respectively). The combination of the two is not feasible in working environments. To address this issue, future research will evaluate other combinations of noninvasive sensors to achieve the same as this study has accomplished. Finally, other noninvasively monitored variables with proven applicability in fatigue estimation approaches (e.g., accelerometry [52]) will also be evaluated.

## 5. Conclusions

This study developed a four-level physical fatigue prediction model using physiological signals and a machine learning approach. XGBoost classifier made the best physical fatigue estimations using 21 features from heart rate, breathing rate, core temperature and personal characteristics. The group cross-validation method gave the most practical view of the model’s performance and determined an 82% accuracy by evaluating it in each of the 24 participants. Although there is room for improvement, this high accuracy proved the feasibility of using these variables and machine learning techniques to monitor fatigue among firefighters. This study contributes with a new alternative for continuous and objective methods for monitoring firefighters’ physical fatigue. Real-time physical fatigue monitoring could enhance firefighters’ health and safety by reducing the possibility of overexertion leading to fatigue, helping to monitor those at a higher risk of physical fatigue development and enabling intervention before any injury or accident occurs. 

## Figures and Tables

**Table 1:** Correspondence of the 6–20 Borg scale with the simplified 4-level physical fatigue scale.

RPE	Level of Exertion	Physical Fatigue Levels
**6**	No exertion	Low
**7**		
**7.5**	Extremely light	
**8**		
**9**	Very light	
**10**		
**11**	Light	
**12**		Moderate
**13**	Somewhat hard	
**14**		
**15**	Hard	Heavy
**16**		
**17**	Very Hard	Severe
**18**		
**19**	Extremely hard	
**20**	Maximal exertion	

**Table 2:** Incremental running protocol measured variables (means ± SD) in each 4 min stage. The variables used for extracting the features and labels of the model are in bold. Superscripts represent values significantly different from noted stages (e.g., ^4–7^, differences in stages 4, 5, 6 and 7).

Stage No.:	1	2	3	4	5	6	7
Treadmill velocity (km/h)	5.4 ± 1.8	6.2 ± 1.9	7.0 ± 2.1	8.0 ± 2.1	9.0 ± 2.1	10.0 ± 2.1	11.0 ± 2.1
Oxygen uptake (mL/min/kg)	18.7 ± 7.2 ^2–7^	22.5 ± 8.3 ^3–7^	25.5 ± 9.2 ^4–7^	29.5 ± 9.4 ^5–7^	34.5 ± 9.1 ^6–7^	39.1 ± 8.6	40.5 ± 8.6
Oxygen uptake (mL/min)	1418.5 ± 571.9 ^2–7^	1688.3 ± 630.3 ^3–7^	1919.3 ± 706.5 ^4–7^	2228.0 ± 731.9 ^5–7^	2611.2 ± 709.4 ^6–7^	2964.5 ± 684.9	3065.2 ± 698.1
**Respiratory frequency (1/min)**	26 ± 7 ^2–7^	28 ± 8 ^4–7^	31 ± 7 ^4–7^	35 ± 7 ^5–7^	40 ± 8 ^6–7^	47 ± 8 ^7^	52 ± 8
Tidal volume (L)	1.25 ± 0.38 ^2–7^	1.47 ± 0.48 ^5–7^	1.50 ± 0.46 ^4–7^	1.65 ± 0.40 ^5–7^	1.86 ± 0.39 ^6–7^	2.03 ± 0.43	1.99 ± 0.34
Ventilation (L/min)	33 ± 14 ^2–7^	42 ± 16 ^3–7^	47 ± 18 ^4–7^	58 ± 18 ^5–7^	75 ± 20 ^6–7^	94 ± 19 ^7^	105 ± 20
Carbon dioxide production (mL/min)	1260.2 ± 533.5 ^2–7^	1601.3 ± 609.4 ^3–7^	1802.1 ± 689.9 ^4–7^	2170.0 ± 708.7 ^5–7^	2641.8 ± 689.9 ^6–7^	3091.8 ± 609.3	3236.2 ± 691.7
Respiratory quotient	0.89 ± 0.13 ^2–7^	0.95 ± 0.15 ^5–7^	0.95 ± 0.17 ^6–7^	0.99 ± 0.20 ^5–7^	1.03 ± 0.20^7^	1.06 ± 0.19	1.07 ± 0.20
**Heart rate (bpm)**	110 ± 17 ^2–7^	127 ± 20 ^3–7^	139 ± 22 ^4–7^	153 ± 20 ^5–7^	168 ± 13 ^6–7^	179 ± 11	183 ± 20
**Core temperature (°C)**	37.36 ± 0.36 ^3–7^	37.47 ± 0.40 ^3–7^	37.56 ± 0.40 ^4–7^	37.71 ± 0.45 ^5–7^	37.81 ± 0.40 ^6–7^	38.01 ± 0.40 ^7^	38.19 ± 0.44
Lactate concentration (mmol/L)	2.4 ± 1.0 ^4–7^	2.6 ± 1.0 ^3–7^	3.1 ± 1.4 ^4–7^	4.3 ± 1.5 ^5–7^	6.0 ± 3.0 ^6–7^	10.3 ± 3.4 ^7^	14.4 ± 4.0
**RPE (6–20)**	9 ± 2 ^2–7^	11 ± 2 ^3–7^	12 ± 2 ^4–7^	14 ± 2 ^5–7^	16 ± 2 ^6–7^	17 ± 1 ^7^	19 ± 1

**Table 3:** Performance metrics for the various classifiers and using three cross-validation methods: (**a**) 10-fold cross-validation, (**b**) stratified 10-fold cross-validation and (**c**) group cross-validation with 24 splits. Bold values show the best score for each performance metric.

Classifier	Accuracy			Precision			Recall			F1 score		
	(a)	(b)	(c)	(a)	(b)	(c)	(a)	(b)	(c)	(a)	(b)	(c)
XGBoosted Tree	**0.88**	**0.88**	**0.82**	**0.89**	**0.88**	**0.82**	**0.88**	**0.88**	**0.82**	**0.88**	**0.88**	**0.82**
Gradient-Boosted Tree	0.87	0.87	0.81	0.87	0.88	0.81	0.87	0.87	0.81	0.87	0.87	0.81
Bagged Tree	0.85	0.84	0.81	0.85	0.84	0.81	0.85	0.84	0.81	0.85	0.84	0.81
Random Forest	0.84	0.84	0.81	0.85	0.85	**0.82**	0.84	0.84	0.81	0.84	0.84	0.81
Linear Support Vector Machine	0.83	0.84	0.77	0.83	0.84	0.77	0.83	0.84	0.77	0.83	0.84	0.77
K-nearest Neighbours	0.80	0.80	0.64	0.80	0.80	0.66	0.80	0.80	0.64	0.80	0.80	0.65
RUSBoosted Trees	0.79	0.79	0.71	0.79	0.80	0.72	0.79	0.79	0.71	0.79	0.79	0.72
Artificial Neural Network	0.73	0.72	0.73	0.72	0.71	0.73	0.73	0.73	0.73	0.72	0.71	0.73
Quadratic Support Vector Machine	0.41	0.42	0.42	0.38	0.43	0.47	0.41	0.42	0.42	0.31	0.31	0.31
Cubic Support Vector Machine	0.45	0.46	0.44	0.46	0.49	0.44	0.45	0.46	0.44	0.36	0.37	0.34
Gaussian Support Vector Machine	0.36	0.38	0.36	0.35	0.46	0.37	0.37	0.38	0.36	0.28	0.29	0.27

## Data Availability

Data is contained within the article.
